# Differentiating Between Lithium Tremors and Extrapyramidal Tremors in a Patient on Long-Term Antipsychotics and Lithium Medication

**DOI:** 10.7759/cureus.42406

**Published:** 2023-07-24

**Authors:** Gurraj Singh, Sherlyne Magny, Harsehaj Singh, Sukhnoor Singh, Inderpreet S Virk

**Affiliations:** 1 Psychiatry, Sri Guru Ram Das Institute of Medical Sciences and Research, Amritsar, IND; 2 Medicine, American University of Integrative Sciences School of Medicine, Bridgetown, BRB; 3 Psychiatry, California Department of Corrections and Rehabilitation, Vacaville, USA

**Keywords:** medication-induced, extrapyramidal symptoms, antipsychotic, lithium, tremor

## Abstract

Tremors are characterized by involuntary rhythmic shaking movement in different regions of the body. Tremors can manifest in various forms and have various causes, including the use of drugs such as lithium and antipsychotic medication. In a clinical setting, it is vital to understand the varieties of tremors presented and administer the appropriate pharmacotherapy needed. We present a case of a patient that has been experiencing fine tremors while on antipsychotics and lithium medication for the past year. We address differentiating the tremors while proposing managing the effects based on their mechanisms of action.

## Introduction

A tremor is a neurological condition characterized by rhythmic shaking movements in different parts of the body. These movements result from involuntary muscle contractions and occur sporadically or continuously [1]. Tremors can manifest in several distinct forms and have multiple causes. These causes include the use of drugs such as lithium, dopamine receptor antagonists (antipsychotics), selective serotonin reuptake inhibitors/serotonin-norepinephrine reuptake inhibitors (SSRIs/SNRIs), amitriptyline, amiodarone, valproate, β-adrenoceptor agonists, and vesicular monoamine transporter 2 (VMAT2) inhibitors. They can also arise from consuming cocaine and ethanol [2]. Other conditions or illnesses that produce tremors include multiple sclerosis, Parkinson's disease, stroke, traumatic brain injury, hypoglycemia, and an overactive thyroid [1].

Lithium-induced tremors are a type of postural tremor that fall under the category of exaggerated physiological tremors [3]. They are observed when a specific posture is voluntarily maintained against gravity and typically exhibit a frequency of 8-12 Hz [4]. Lithium-induced tremors typically manifest early during treatment, although they can appear at any point in time. The prevalence of lithium tremors tends to be higher in older individuals, potentially due to the cumulative impact of age-related essential tremors [5].

Tremors caused by extrapyramidal symptoms (EPS) are involuntary rhythmic movements of the hands, arms, mouth-face, or other body parts that can occur as a side effect of some drugs, notably antipsychotics. The disruption of the basal ganglia's normal functioning is the underlying mechanism of action for EPS tremors [6]. A separate study found that among 144 cases, the prevalence of EPS was 50.5%, with tremors being the predominant EPS observed in 94 cases and frequently co-occurring with other forms [7]. Parkinsonism was the second most common, with 38 cases [7].

We present a case of a patient that has been experiencing fine tremors for the past year while on aripiprazole and lithium. We address differentiating between lithium tremors and extrapyramidal tremors while proposing managing the effects based on their mechanisms of action.

## Case presentation

A 56-year-old female with a history of schizoaffective disorder-bipolar type presented to the outpatient clinic for medication management. The patient reported being psychiatrically stable while taking aripiprazole 20 mg orally (PO) daily and lithium 300 mg PO three times a day (TID) since starting two years ago, with her last lithium levels confirmed at 0.6 mEq/L one month before the visit. The patient mentioned no significant side effects from her medication but expressed having fine tremors in her hands starting one year ago. However, at the time, her tremors were causing mild to moderate distress with minimal effect on her daily activity. She also endorsed lithium being life-changing and not wanting to adjust her dosage.

The patient's psychiatric history started in her 20s, with multiple manic/depressive episodes with psychotic symptoms. Based on her timeline and symptomatology, she met the criteria for schizoaffective disorder-bipolar type. Additionally, she had been hospitalized four times, with the last hospitalization seven years ago for trying to commit suicide by overdosing on pills and jumping in front of oncoming traffic. Past medications include olanzapine, quetiapine, risperidone, lurasidone, fluoxetine, escitalopram, and sodium valproate. The patient felt that being on aripiprazole and lithium provided her with the most effective treatment for managing her symptoms.

The patient had a past substance dependence history of crack/heroin/meth use (non-IV) but has been abstinent for seven years without cravings. She also denied any history of alcohol abuse. She reported quitting smoking cigarettes three years back but relapsed six months ago and smoked one pack daily. The patient was encouraged to start on a nicotine patch and, as needed, nicotine lozenges to help her with smoking cessation.

Before the upcoming appointment, laboratory investigations were obtained, including complete blood count (hemoglobin {Hb}: 13.8 g/dL), chemistry metabolic panel, thyroid-stimulating hormone (TSH) (1.9 mIU/L), and lithium levels (0.6 mEq/L), which were unremarkable. Lithium levels were found unchanged.

Considering the patient's history, current medications, and ongoing symmetrical fine tremors of the hand, the tremors were believed to be lithium-induced, despite normal lithium levels. Tremors induced by antipsychotics were ruled out based on no additional EPS, including involuntary truncal, limb, and oral-facial movements. Additionally, the patient was put on benztropine in the past with no response to her tremors. It was discussed and considered with the patient to start propranolol for the fine tremors for possibly being lithium-induced. At the time, the patient expressed disinterest in adding further medication. She remained on aripiprazole 20 mg PO daily for mood stabilization and psychosis management. Lithium 300 mg PO TID was unchanged for mood stabilization with normalized lithium levels.

At the patient's first one-month follow-up, she reported doing well with no worsening psychiatric symptoms. However, she had noticed that her fine hand tremors began to worsen and bothered her during day-to-day activities, for example, causing difficulty in eating and writing. The patient agreed to start propranolol 10 mg PO twice per day (BID) for the tremors while continuing aripiprazole 20 mg PO and lithium 300 mg PO TID. The patient was also encouraged to quit smoking cigarettes. By this time, the patient had reduced smoking to half a pack from one pack a day, as the patient found the nicotine patches and gum helpful, which were continued. The patient was reported stable.

At the patient's second one-month follow-up, she reported significant improvement in her hand tremors without experiencing any side effects from propranolol. The propranolol morning dose was increased to 20 mg while keeping the evening dose of 10 mg the same and remaining on aripiprazole 20 mg PO and lithium 300 mg PO TID. The patient reported no additional side effects from medication, with no change in the intensity of symptoms or any new onset of symptoms.

During the patient's third and most recent follow-up, her hand tremors resolved entirely, emphasizing how propranolol helped her tremors while allowing her to continue her daily activities without disruption. She expressed no side effects, change in intensity of symptoms, or any new onset of symptoms from her medications. By this time, the patient had stopped smoking and reported doing well overall.

## Discussion

Drug-induced tremors are among the common involuntary movement disorders seen in clinical practice. These tremors are unintentional adverse effects noticed in antipsychotics and mood stabilizers such as lithium carbonate. Drug-induced tremors often have benign consequences; however, when the tremors become bothersome to the patient's lifestyle, then treatment intervention is warranted. This case highlights the importance of differentiating the characteristics of tremors as a recommendation for clinicians to determine the possible causes of these involuntary movements.

Dopamine-receptor-blocking agents, antipsychotics, are the most common medications associated with extrapyramidal movement disorders [8]. The movement disorders usually involve symptoms that include restlessness, involuntary muscle contraction, tremors, stiff muscles, and involuntary facial movements [6]. Antipsychotics that induce tremors can vary from acute manifestations, such as dystonia, akathisia, and parkinsonism, to severe symptoms, such as tardive akathisia and tardive dyskinesia [6]. These manifestations are debilitating, interfering with speech and daily tasks. Without treatment and intervention, this is often associated with poor quality of life and may prevent patients from continuing therapy. It is essential to mention that extrapyramidal symptoms that developed in schizophrenic patients are associated with poor compliance with other atypical antipsychotic medications [8]. Thus, clinicians must determine whether the patients are developing movement disorders due to stopping pharmacologic therapy.

Lithium-induced tremors are usually characterized as symmetrical fine hand tremors. Unlike antipsychotic-induced tremors, the tremors classically present as postural tremors [9]. To clarify, acute lithium tremors are observed when the hand is at rest and the tremor increases with voluntary movements, such as writing or holding a coffee cup [9]. This well-known adverse effect may indicate the early signs of toxicity even when the patients are on low therapeutic doses [9,10]. In this case, our patient presented with fine hand tremors that worsened during her daily activities, such as writing, while her lithium level was ideal for bipolar disorder.

Moreover, antipsychotic-induced tremors were ruled out in our patient due to no EPS observed, including involuntary truncal, limb, and oral-facial movements, while having a history of using benztropine with no relief. This was confirmed with tremor improvements while on beta-blockers, propranolol, the standard management to alleviate lithium-induced tremors. Clinicians must consider that a little over half of the patients starting on lithium therapy develop tremors during the first week [11], but tremors can occur at any time during treatment. Therefore, the anticipated side effect should be discussed with the patients. Thus, monitoring lithium levels is encouraged, and observing tremor characteristics and its treatment responses may differentiate other potential drug causes.

Distinguishing drug-induced tremors with their commonly associated symptoms aided in determining whether it was lithium- or antipsychotic-related. For instance, parkinsonism, an adverse effect seen in antipsychotic medications, may present with clinical characteristics such as bilateral symmetrical tremors at rest and postural tremors [12]. Although these features are similar to lithium-induced tremors, asymmetrical hand tremors, bradykinesia, limb stiffness, mask-like facies, and salivation are major associated features of parkinsonism [12,13]. Furthermore, since bilateral postural tremors of the hands and fingers were the only evident symptom our patient experienced without any other abnormal movement patterns present, we excluded antipsychotic causes.

In addition, peak frequency may be studied to differentiate drug-induced tremors. Lithium tremors share some clinical characteristics of parkinsonism tremors, so electromyography can be a valuable tool for assessing tremor patterns when clinical diagnoses are not straightforward. The accelerometric tremor recordings in parkinsonism tremors are usually higher in amplitude and have a low peak frequency between 4 and 7 Hz compared to lithium tremors [11,14]. Lithium tremors can be detected to have a significantly higher peak frequency with an average of 8 Hz [14]. For the patients experiencing debilitating tremors, using an electromyography may be a necessary step in diagnosis.

Risk factors have been identified with drug-induced movement disorders. Lithium-induced tremors are usually seen in the elderly, with toxic lithium levels, preexisting antidepressant usage, excessive caffeine intake, personal or family history of tremors, alcohol withdrawal, and anxiety [11,15]. Thus, clinicians need to be aware of the risk factors when starting a patient on lithium and monitor lithium levels. It is also necessary for clinicians to have a risk-benefit discussion with the patient and encourage patients to avoid any potential factors that may cause tremors in general.

Antipsychotic-induced tremors are classically seen in those with increasing age and tremor history and smokers [16]. Smoking reduces the risk of developing extrapyramidal movement disorders arising from antipsychotic medications due to increased hepatic drug metabolism by activating the enzymes of the cytochrome P450 enzymatic family [16]. Our patient smoked for many years before developing hand tremors, making the antipsychotics' adverse effects not likely. Most importantly, the patient's tremors were significantly treated by the first follow-up only after she was given propranolol, making the use of lithium the more likely cause.

## Conclusions

Lithium-induced tremors can be challenging to recognize and diagnose, especially when their presentation can be indistinguishable from parkinsonism movement disorders due to antipsychotics. However, antipsychotic-induced tremors are usually associated with other clinical features such as involuntary truncal, limb, and oral-facial movements. With parkinsonism, it is usually presented with mask-like faces, limb rigidity, and tremors. Moreover, examining drug-induced tremors using electrophysiological examination may provide valuable diagnostic information due to the distinct characteristics in frequency and amplitude when contrasting lithium and parkinsonism tremors. Clinicians should consider possible factors that may worsen tremors, such as caffeine, anxiety, or withdrawals. Furthermore, regular lithium level checks to avoid neurotoxicity, although inevitable at subtherapeutic doses, are vital to minimize tremor development. Nevertheless, if the inevitable does occur, beta-blockers are the current treatment for lithium-induced tremor.
